# Low vitamin D levels in post-menopausal women are associated with complex regional pain syndrome type I in surgically treated distal radius fractures

**DOI:** 10.1186/s13018-020-01859-4

**Published:** 2020-08-14

**Authors:** Sang-Uk Lee, Ki-Tae Na, Yoon-Min Lee, Jong Hwa Park, Sun Young Joo

**Affiliations:** 1 Department of Orthopedic Surgery, Incheon St. Mary’s Hospital, The Catholic University of Korea, 56 Dong-su ro, Bupyeong-gu, Incheon, 21431 Republic of Korea; 2 Department of Orthopedic Surgery, Yeouido St. Mary’s Hospital, The Catholic University of Korea, Seoul, Korea

**Keywords:** Vitamin D, Complex regional pain syndrome, Distal radius fracture

## Abstract

**Background:**

Complex regional pain syndrome type I (CRPS I) is a chronic devastating condition and a relatively common complication of distal radius fractures (DRF). The purpose of this study was to investigate the relationship of vitamin D levels in surgically treated post-menopausal women with CRPS I occurrence in DRF.

**Methods:**

From February 2016 to March 2017, 158 surgically treated post-menopausal patients with DRF were enrolled. Exclusion criteria were (1) patients who had been taking vitamin D or osteoporosis medication at the time of injury; (2) patients with medical factors that may affect vitamin D levels; (3) patients who were reluctant to enroll in the study; and (4) patient with additional fractures, ligamentous injuries, or neuropathy. A total of 107 patients were available for final analysis. We compared the serum vitamin D levels in post-menopausal women with DRF with CRPS I (group 1) and without CRPS I (group 2). Bone mineral density (BMD) of the femur and spine, osteocalcin, alkaline phosphatase (ALP), body mass index (BMI) were also measured.

**Results:**

The average age at the time of surgery was 66.5 years (range, 39-86 years). The mean follow-up period was 16.3 months after surgery. Among the 107 surgically treated DRF patients, 19 (18%) met the Budapest criteria for CRPS I during the follow-up period. The mean serum vitamin D level in group 1 (15.2 ng/ml) was significantly lower than that in group 2 (20.5 ng/ml, *p* = 0.027). The mean values of osteocalcin, ALP, BMI, and BMD were not significantly different between the groups.

**Conclusion:**

Lower vitamin D levels in post-menopausal women can increase CRPS I occurrence in distal radius fractures.

## Background

Complex regional pain syndrome type I (CRPS I) is a chronic devastating condition involving the limbs, characterized by unexplained severe pain that is constant, extremely intense, and out of proportion to the original injury. It is also accompanied by swelling, autonomic dysfunction, and joint stiffness. CRPS I is known to occur more frequently in the upper extremities of female patients between 61 and 70 years of age [1–4]. Fractures appear to be a common inciting event for the development of CRPS I. Common fractures with CRPS I include ankle, tibia, and distal radius fractures (DRF).

Patients with DRF are at high risk for developing CRPS I. The incidence of CRPS I after DRF varies widely between studies, and is reported to range from 1 to 37%, which is relatively higher than other fractures [2, 5–8]. Prognosis of CRPS I in DRF is good if treated early and properly [2]; however, it becomes poor if managed late. It also exerts a large financial burden on the healthcare system and is a common cause of lawsuits [9].

The occurrence of CRPS I causes severe pain with adverse psychosocial and socioeconomic effects on the patient’s quality of life and daily function [10]. As a result, patients with CRPS I are unable to actively participate in rehabilitation, have a delayed recovery, and a higher probability of poorer final clinical outcomes. Therefore, the prevention of this condition is important for both surgeons and patients.

Several studies have evaluated the risk factors for CRPS I development in DRF patients; however, there has been no consensus because of the heterogeneity of CRPS I diagnosis and treatment methods [9, 11, 12]. Zollinger et al. treated 416 conservatively or surgically treated DRF patients where only plaster and vitamin C therapy were associated with the development of CRPS I [13]. Demir et al. revealed the association between female gender, motor nerve injury, and CRPS I in 165 conservatively or surgically treated DRF patients [14]. Beerthuizen et al. concluded that intra-articular fracture, dislocations, and musculoskeletal comorbidities were associated with the occurrence of CRPS I in patients with DRF [5]. To the best of our knowledge, the pathophysiology of CRPS I in patients with DRF has not yet been established, and the risk factors have not been clearly identified.

Among the several possibilities, an inflammatory response to trauma is regarded as a reliable pathophysiology for the acute phase of CRPS I. Initial trauma triggers the release of pro-inflammatory cytokines and neuropeptides [15, 16]. As a result, the clinical findings of the acute phase of CRPS I are considered to be the result of inflammation [17].

Because of the anti-inflammatory nature of vitamin D [18–22], we hypothesized that a lower vitamin D level can result in increased occurrence of CRPS I in DRF patients. The main function of vitamin D is to regulate homeostasis of calcium and phosphorus. It also plays a central role in mineralization, growth, and remodeling of bone. Vitamin D deficiency correlates with decreased bone density and rigidity in children (rickets). Moreover, a recent study showed additional vitamin D functions that include modulation of muscle function, inflammation, and the immune system [19–22]. Vitamin D has also been shown to significantly downregulate cellular response to tumor necrosis factor (TNF)-α, interleukin (IL)-6, and C-reactive protein (CRP).

The primary goal of the current study is to prospectively compare serum vitamin D levels in surgically treated post-menopausal DRF patients with and without CRPS I and to analyze the influence of vitamin D and other factors on the incidence of CRPS I after DRF.

## Patients and methods

### Study design

We conducted a retrospective study to investigate the relationship between the occurrence of CRPS I and vitamin D levels among surgically treated DRF patients. The institutional review board of our hospital approved the study. We included menopaused female patients who underwent surgery for DRF and in whom we were able to assess radiographic and clinical data for more than 1 year postoperatively. All surgeries were performed at a single institution by two seasoned surgeons from February 2016 to March 2017.

We identified 158 women with a DRF from February 2016 to March 2017. Exclusion criteria were as follows: (1) patients who had been taking vitamin D or osteoporosis medication at the time of injury (24 pts); (2) patients with medical factors that may affect vitamin D levels, such as gastrointestinal or renal disease (14 pts); (3) patients who were reluctant to enroll in the study (8 pts); and (4) patients with additional fractures, ligamentous injuries, or neuropathies (5 pts). Thus, 107 patients were included in this study.

Patients who had CRPS I were defined as “Group 1” while those who did not were identified as “Group 2.” The average age at the time of surgery was 66.5 years (range, 39-86 years). The right upper extremity was affected in 50 cases and the left side was affected in 57 cases. The dominant upper extremity was involved in 63 cases (right, 49; left, 14). The mean follow-up period was 16.3 months after surgery (range, 12-25 months). According to the AO classification of DRF, there were 4 with type A2, 20 with type A3, 5 with type B2, 1 with type B3, 5 with type C1, 42 with type C2, and 30 with type C3.

### Diagnosis of CRPS I

CRPS I was diagnosed using the Budapest diagnostic criteria for research (Table 1), which is a modified criterion of the International Association for the Study of Pain (IASP, 2007) [23]. A diagnosis of CRPS I was made when a patient showed the symptoms and fulfilled at least 2 of the criteria. The patients were assessed for symptoms and signs of CRPS I by the orthopedic hand specialists at 4 weeks, 8 weeks, 12 weeks, 16 weeks, and 20 weeks postoperatively, and at 2-month intervals after 20 weeks. To confirm the diagnosis, a patient who was suspected of having CRPS I by a physician was referred to another physician to confirm the diagnosis once more. The diagnosis of CRPS I was confirmed when two hand surgeons agreed to the diagnosis.

**Table 1:** Budapest diagnostic criteria for CRPS (for research purpose)

1. Continuing pain, which is disproportionate to any inciting event 2. Must report at least one symptom in all four following categories: • Sensory: reports of hyperesthesia and/or allodynia • Vasomotor: reports of temperature asymmetry and/or skin color changes and/or skin color asymmetry • Sudomotor/edema: reports of edema and/or sweating changes and/or sweating asymmetry • Motor/trophic: reports of decreased range of motion and/or motor dysfunction (weakness, tremor, dystonia) and/or trophic changes (hair, nail, skin) 3. Must display at least one sign at the time of evaluation in two or more of the following categories: • Sensory: evidence of hyperalgesia (to pinprick) and/or allodynia (to light touch and/or temperature sensation and/or deep somatic pressure and/or joint movement) • Vasomotor: evidence of temperature asymmetry (> 1) and/or skin color changes and/or asymmetry • Motor/trophic: evidence of decreased range of motion and/or motor dysfunction (weakness, tremor, dystonia) and/or trophic changes (hair, nail, skin) 4. There is no other diagnosis that better explains the signs and symptoms	

### Treatment strategy

Among the 107 surgically treated DRF patients, three different treatment strategies were applied depending on the type of fracture: 94 patients (88%) underwent open reduction and internal fixation with a volar locking plate; 6 patients (6%) with severe dorsal comminution (more than 50% of articular surface) underwent operation with a dorsal plate; and 7 patients (6%) with severe articular comminuted fracture were treated with external fixation and Kirschner wires or plate fixation at the same time.

The external fixators and additional Kirschner wires were removed at 2 weeks postoperatively in patients who had plate fixation. For those without an external fixator, the sugar tong splint was applied for 2 weeks, postoperatively. After that, we applied a removable wrist brace and gentle passive range of motion exercises were introduced. Physical therapy was continued for another 2 months postoperatively.

### Laboratory evaluation

Although 1,25(OH)_2_D_3_ is the biologically active form of vitamin D, we measured 25(OH) D_3_ (calcifediol) levels in the present study because its serum concentration is 1000 times that of 1,25(OH)_2_D_3_, and it reflects the actual status of vitamin D level of the serum more accurately, especially in the population aged > 40 years [24]. All blood samples were taken during fasting, and patients were sampled during the preoperative evaluation. The level of 25(OH)D_3_ was measured with a chemiluminescence immunoassay (Adiva Centaur, Siemens Healthineers, Erlangen, Germany). All assays were performed at the department of laboratory medicine at our institution, which was blind to the study. The time between blood sample collection and occurrence of DRF did not exceed 10 days. In the current study, vitamin D sufficiency was defined as a serum 25(OH)D_3_ level > 32 ng/ml; insufficiency as a serum level from 20 to 32 ng/ml; and deficiency as a serum level of < 20 ng/ml [25].

### Statistical analysis

All statistical analyses were performed using the R language ver. 3.3.3 (R Foundation for Statistical Computing, Vienna, Austria), and T & F program ver. 2.0 (YooJin BioSoft, Korea), and a *p* value < 0.05 was determined as the level of statistical significance. When continuous variables were normally distributed, a mean difference test between the two groups was performed using the Student *T* test or Welch’s *T* test. For non-normally distributed variables, a Mann-Whitney *U* test was used. For categorical variables, a chi-squared test or Fisher’s exact test was performed to test the hypothesis of the association between CRPS I and other variables as appropriate using a contingency table. Receiver operating characteristic (ROC) curve analysis was performed to estimate the prediction accuracy of continuous values on the binary response defined by CRPS I. Binary logistic regression analysis was performed to analyze the effect of each clinical measurement on the binary response of CRPS I. For analyzing the combined effect of more than two variables on the CRPS I response, a multivariable logistic regression analysis was performed using a backward stepwise procedure as the variable selection method to minimize Akaike Information Criterion (AIC). A significance level of 0.1 was used in the univariable analysis to select initial input variables for the multivariable analysis. Prediction performance was estimated by several measures such as AIC, Nagelkerke R2, rank correlation, and C-index.

## Results

Among the 107 surgically treated DRF patients, 19 (18%) met the Budapest criteria for CRPS I during the follow-up period. The number of patients who were newly diagnosed with CRPS I was 14, 3, 2, 0, and 0 at 4, 8, 12, 16, and 20 weeks after surgery, respectively. The number of patients who met the diagnostic criteria at each occasion were 14, 14, 6, 2, and 0 at 4, 8, 12, 16, and 20 weeks after surgery, respectively. Among 14 patients who were initially diagnosed with CRPS I at 4 weeks, symptoms and signs of CRPS I were persistent at 8 weeks postoperatively in 11 patients and at 12 weeks in 2 patients; no patients had any symptoms or signs at 16 weeks. Among 3 patients who were initially diagnosed with CRPS I at 8 weeks, symptoms and signs of CRPS I were persistent at 12 weeks postoperatively in 2 patients and at 16 weeks postoperatively in 1 patient. Among two patients who were initially diagnosed with CRPS I at 12 weeks, symptoms and signs of CRPS I were persistent at 16 weeks in 1 patient. No patient met the diagnostic criteria for CRPS I at 20 weeks postoperatively. The mean period of suffering for CRPS I was 7.6 weeks (range, 4-12 weeks). The mean time from surgery to occurrence of CRPS I was 5.4 weeks. (range, 4-12 weeks).

Table 2 compares the mean values of the various items between groups. The mean value of serum 25(OH)D_3_ level in group 1 was significantly lower than that in group 2. The mean age of group 1 was significantly younger than that of group 2 (*p* = 0.023). The mean values of osteocalcin, ALP, BMI, and BMD of the femur and spine were not significantly different between the groups.

**Table 2:** Mean comparison analysis between two groups

Variable	Mean values (mean ± SE)			*p* value
	Total	DRF with CRPS I (group 1)	DRF without CRPS I (group 2)	
*N*	107	19	88	
Age (years)	67.3 ± 0.9	62.7 ± 2.0	68.3 ± 1.0	0.023
25(OH)D_3_ (ng/ml)	19.6 ± 0.9	15.2 ± 1.7	20.5 ± 1.0	0.027
Osteocalcin (ng/ml)	23.4 ± 2.3	21.1 ± 1.9	24.0 ± 2.8	0.628
ALP (U/l)	93.6 ± 3.7	91.2 ± 10.6	94.1 ± 3.9	0.458
BMI (kg/m^2^)	24.1 ± 0.3	23.9 ± 0.8	24.1 ± 0.3	0.758
BMD (femur)	−1.9 ± 0.1	−1.6 ± 0.2	−1.9 ± 0.1	0.301
BMD (spine)	−2.4 ± 0.1	−2.6 ± 0.3	−0.6 ± 1.2	0.645

*SE* standard error, *DRF* distal radius fracture, *CRPS I* complex regional pain syndrome type I, *25(OH) D*_*3*_ 25-hydroxyvitamin D_3_, *ALP* alkaline phosphatase, *BMI* body mass index, *BMD* bone mineral density

Table 3 shows the association analysis between vitamin D level and CRPS I. Among 107 patients, 94 (88%) were included based on vitamin D insufficiency or deficiency criteria, and only 13 (12%) were included based on vitamin D sufficiency criteria. Although the difference was not statistically significant (*p* = 0.547), the incidence of CRPS I tended to decrease as vitamin D levels increased from deficiency (22%) to insufficiency (15%) and sufficiency (8%).

**Table 3:** Association analysis between vitamin D level and CRPS I

25(OH)D_3_	N (%)	Group 1	Group 2	OR (95% CIs)
		*N* (%)	*N* (%)	
Sufficiency	13 (100)	1 (8)	12 (92)	0.301 (0.036-2.536)
Insufficiency	34 (100)	5 (15)	29 (85)	0.623 (0.201-1.931)
Deficiency	60 (100)	13 (22)	47 (78)	1

*p* value = 0.547; *p* value was computed using Fisher’s exact test

*25(OH)D*_*3*_ 25-hydroxyvitaminD_3_, *DRF* distal radius fracture, *CRPS* complex regional pain syndrome type I, *OR* odd ratio, *95% CIs* 95% confidence intervals, *sufficiency* serum 25(OH)D_3_ level > 32 ng/mL, *insufficiency* serum 25(OH)D_3_ level from 20 to 32 ng/mL, *deficiency* serum 25(OH)D_3_ level < 20 ng/mL 0.623 (0.201-1.931)

Table 4 shows the mean comparison of seasonal vitamin D level and association analysis between season and CRPS I. Although there was no statistically significant difference in the mean level of 25(OH)D_3_ between seasons, the risk of developing CRPS I was higher in the spring (32%) than in other seasons (14%) (*p* = 0.322).

**Table 4:** Mean comparison analysis of seasonal vitamin D level and association analysis between season and CRPS I

Season	No. of patient (%)	Serum 25(OH)D_3_ (mean ± SE, ng/mL)	No. of CRPS patients (%)	OR (95% CIs)
Spring	22 (100)	19.6 ± 1.8	7 (32)	1
Summer	18 (100)	19.7 ± 2.5	3 (17)	0.429 (0.093-1.98)
Fall	29 (100)	19.0 ± 1.7	4 (14)	0.343 (0.086-1.37)
Winter	38 (100)	19.9 ± 1.7	5 (13)	0.325 (0.088-1.191)
*p* value		0.988*	0.322†	

*DRF* distal radius fracture, *CRPS I* complex regional pain syndrome type I, *OR* odd ratio, *95% CIs* 95% confidence intervals

*p value: mean comparison analysis among 4 subgroups of season, computed using 1-way ANOVA

†*p* value: association analysis between CRPS I and seasons, computed using Fisher’s exact test

The occurrence of CRPS I in DRF patients was related to serum 25(OH) D_3_ level and age in the univariable binary logistic regression analysis (Table 5). Other factors such as ALP, osteocalcin, BMI, and BMD did not influence the occurrence of CRPS I in the current study. AO classification and use of an external fixator did not increase the risk of CRPS either. The multivariable binary regression analysis also showed serum 25(OH)D_3_ level and age to be independent variables for the occurrence of CRPS I in DRF patients (Table 6). Since the C index of the current multivariable binary logistic regression analysis was 0.778, the prediction performance of the current model is good (C index = 1 indicates prediction perfection).

**Table 5:** Results of univariable binary logistic regression analysis using CRPS I as response

Predictor	Subgroup	OR (95% CIs)	*p* value
Vitamin D	Serum 25(OH) D_3_ level	0.929 (0.868-0.994)	0.032
	Sufficiency vs. insufficiency vs. deficiency		0.439
	Sufficiency vs. deficiency	0.301 (0.036-2.536)	0.27
	Insufficiency vs. deficiency	0.623 (0.201-1.931)	0.413
ALP		0.998 (0.984-1.012)	0.762
Osteocalcin		0.987 (0.937-1.039)	0.623
Age		0.937 (0.883-0.993)	0.028
Season			0.302
	Summer vs. spring	0.429 (0.093-1.98)	0.278
	Fall vs. spring	0.343 (0.086-1.37)	0.13
	Winter vs. spring	0.325 (0.088-1.191)	0.09
	summer and fall vs. winter and spring	0.7 (0.252-1.946)	0.494
AO classification			0.192
	B vs. A	9.2 (0.692-122.351)	0.093
	C vs. A	6.133 (0.769-48.914)	0.087
External fixation			0.723
	EF with K wires vs. ORIF with plate	2.441 (0.209-28.475)	0.476
	EF with plate vs. ORIF with plate	1.627 (0.16-16.603)	0.681
Dorsal plating		2.471 (0.418-14.587)	0.318
BMI		0.975 (0.834-1.141)	0.756
BMD (femur)		1.341 (0.771-2.331)	0.298
BMD (spine)		0.902 (0.583-1.394)	0.641

*OR* odd ratio, *95% CIs* 95% confidence intervals, *25(OH)D*_*3*_ 25-hydroxyvitaminD_3_, *ALP* alkaline phosphatase, *EF* external fixation with external fixator, *K wires* Kirschner wires, *ORIF* open reduction and internal fixation, *BMI* body mass index, *BMD* bone mineral density

**Table 6:** Result of multivariable binary logistic regression analysis using CRPS I as response

Predictor	Subgroup	OR (95% CIs)	*p* value
Vitamin D	serum 25(OH) D_3_ level	0.932 (0.876-0.992)	0.03
Age		0.921 (0.856-0.992)	0.026
AO classification			0.231
	B vs. A	5.959 (0.382-93.01)	0.203
	C vs. A	6.265 (0.765-51.344)	0.087

*CRPS I* complex regional pain syndrome, *OR* odd ratio, *95% CIs* 95% confidence intervals, *25(OH)D*_*3*_ 25-hydroxyvitaminD_3_

## Discussion

We aimed to compare serum vitamin D levels in DRF patients with and without CRPS I. We also evaluated the influence of vitamin D and other factors on the incidence of CRPS I after DRF.

The most important finding was the statistical difference in the mean serum 25(OH)D_3_ level between the groups in the current study. The univariate and multivariate binary logistic regression analysis showed a statistically significant relationship among younger age, low serum 25(OH)D_3_ level, and the occurrence of CRPS I after DRF. Recent study shows that vitamin D deficiency is associated with neuropathic pain. Yesil et al. reported that in patients with rheumatoid arthritis, the prevalence of neuropathic pain was 5.8 times higher among patients with serum vitamin D levels below 20 ng/mL compared to patients with vitamin D values 30 ng/mL or over [26]. To the best of our knowledge, there have been no studies on the association of serum vitamin D levels with the incidence of CRPS I in patients with distal radius fracture, our findings showed vitamin D influences the occurrence of CRPS I.

Vitamin D deficiency affects approximately 1 billion people worldwide [27]. Many people, especially the elderly, suffer from vitamin D deficiency due to increased indoor living [28, 29], which correlates with decreased bone density and rigidity, as well as adverse effects on muscle health and healing [30, 31].

The pathophysiology of CRPS I remains unclear; however, clinical manipulations of the acute phase of CRPS I, pain, swelling, redness with vasomotor instability, and increased temperature and impaired function, support the hypothesis that the development of this condition is due to an exaggerated inflammatory response to trauma [17]. Tissue trauma triggers the release of pro-inflammatory cytokines such as IL-1β, IL-2, IL-6, and TNF-α along with neuropeptides including calcitonin gene-related peptide, bradykinin, and substance P. These substances increase plasma extravasation and vasodilation, producing the characteristic features of acute CRPS. Our hypothesis was that the anti-inflammatory function of vitamin D may affect the occurrence of CRPS I in DRF patients. Recently, vitamin D has been shown to significantly downregulate cellular response to TNF-α, a major inflammatory cytokine [21]. Moreover, other proinflammatory cytokines, IL-6 and CRP, were lower in patients who had sufficient serum vitamin D [32]. This anti-inflammatory effect can explain how vitamin D is effective in preventing CRPS I in DRF patients. However, more research is needed to delve into the mechanisms of vitamin D as a factor in CRPS I.

CRPS occurs most frequently in patients in their 60s and demonstrates a female predilection, affecting three times more females than males [1]. In many other studies, however, age is not reported as a risk factor for the development of CRPS I [5, 11–14]. Zollinger et al. reported a higher risk of CRPS I in elderly patients (64.5 vs 57.7, *p* = 0.008) [8]. However, in the logistic-regression analysis of the predictive factors, age was not found to be a predictive factor. Roh et al. reported a higher mean age in the group with CRPS (56.9 vs 49.9, *p* = 0.02) [12]. Their univariate model showed that older age was a potential risk factor for CRPS I. In this study, paradoxically, the mean age of group 1 was 5.6 years younger than that of group 2 (62.7 vs. 68.3 years, *p* = 0.023). Between the two studies, our study differs in that the subjects were only postmenopausal women and the mean age of included patients was higher (67.3 vs. 50.5 years). The reason for these completely different results among studies is not yet known. We may assume cautiously that age itself may not be a risk factor for CRPS due to these conflicting results and recommend further study.

Previous studies on the type of fracture and use of an external fixator as risk factors on developing CRPS after DRF showed inconsistent results [9, 11, 12]. Roumen et al. reported that there was no correlation between the development of CRPS I and the fracture type or anatomical end result [9], whereas Bickerstaff et al. evaluated 274 conservatively treated patients and concluded that patients with CRPS I had more severe type and displacement of fractures [11]. Roh et. al. also concluded that high energy injury, severe fractures, and the female gender contribute to the development of CRPS I after the surgical treatment of DRF [12]. In the present study, AO classification and use of an external fixator were not a risk factor in developing CRPS, which is consistent with the findings by Roumen et al. [9].

Our study has some limitations. First, due to its retrospective nature, we were not able to assess all confounding factors which may have affected the results. Second, the study subjects were ethnic Koreans. Korean women are believed to be at risk for vitamin D deficiency because of the high latitude at which they reside (34 to 38° N), lack of vitamin D-fortified foods, reduced outdoor activity, and increased use of sunscreen. Therefore, our results may not be representative of all women with DRF. Third, the exact pathophysiological relationship between CRPS and vitamin D level has not been explained. Furthermore, the etiology of CRPS is multifactorial rather than unifactorial, but the scope of the present study is limited to vitamin D deficiency as the cause. Finally, the follow-up period of patients was just longer than 1 year. However, CRPS in DRF usually occurs from 6 weeks to 6 months after fracture. Therefore, we think that 1 year is sufficient in evaluating the relationship between CRPS and vitamin D.

## Conclusions

In conclusion, lower vitamin D levels may associate with occurrence of CRPS I in post-menopausal women with DRF. Future investigation should focus on the pathomechanism of vitamin D deficiency and CRPS as well as whether vitamin D supplementation can prevent the development of CRPS in patients with DRF.

## Data Availability

Not applicable

## Abbreviations

CRPS I: Complex regional pain syndrome type I
DRF: Distal radius fractures
BMD: Bone mineral density
ALP: Alkaline phosphatase
BMI: Body mass index
TNF: Tumor necrosis factor
IL: Interleukin
CRP: C-reactive protein
